# Spread of the florfenicol resistance *floR* gene among clinical *Klebsiella pneumoniae* isolates in China

**DOI:** 10.1186/s13756-018-0415-0

**Published:** 2018-11-01

**Authors:** Junwan Lu, Jinfang Zhang, Lei Xu, Yabo Liu, Pingping Li, Tingyuan Zhu, Cong Cheng, Shunfei Lu, Teng Xu, Huiguang Yi, Kewei Li, Wu Zhou, Peizhen Li, Liyan Ni, Qiyu Bao

**Affiliations:** 1 School of Medicine and Health, Lishui University, Lishui, 323000 China; 2 School of Laboratory Medicine and Life Sciences/Institute of Biomedical Informatics, Wenzhou Medical University, Wenzhou, 325035 China; 3 The Second Affiliated Hospital, Wenzhou Medical University, Wenzhou, 325035 China

**Keywords:** Florfenicol, *floR*, *Klebsiella pneumoniae*, Plasmid, Human pathogen

## Abstract

**Background:**

Florfenicol is a derivative of chloramphenicol that is used only for the treatment of animal diseases. A key resistance gene for florfenicol, *floR*, can spread among bacteria of the same and different species or genera through horizontal gene transfer. To analyze the potential transmission of resistance genes between animal and human pathogens, we investigated *floR* in *Klebsiella pneumoniae* isolates from patient samples. *floR* in human pathogens may originate from animal pathogens and would reflect the risk to human health of using antimicrobial agents in animals.

**Methods:**

PCR was used to identify *floR*-positive strains. The *floR* genes were cloned, and the minimum inhibitory concentrations (MICs) were determined to assess the relative resistance levels of the genes and strains. Sequencing and comparative genomics methods were used to analyze *floR* gene-related sequence structure as well as the molecular mechanism of resistance dissemination.

**Results:**

Of the strains evaluated, 20.42% (67/328) were resistant to florfenicol, and 86.96% (20/23) of the *floR*-positive strains demonstrated high resistance to florfenicol with MICs ≥512 μg/mL. Conjugation experiments showed that transferrable plasmids carried the *floR* gene in three isolates. Sequencing analysis of a plasmid approximately 125 kb in size (pKP18–125) indicated that the *floR* gene was flanked by multiple copies of mobile genetic elements. Comparative genomics analysis of a 9-kb transposon-like fragment of pKP18–125 showed that an approximately 2-kb sequence encoding *lysR*-*floR*-*virD2* was conserved in the majority (79.01%, 83/105) of *floR* sequences collected from NCBI nucleotide database. Interestingly, the most similar sequence was a 7-kb fragment of plasmid pEC012 from an *Escherichia coli* strain isolated from a chicken.

**Conclusions:**

Identified on a transferable plasmid in the human pathogen *K. pneumoniae*, the *floR* gene may be disseminated through horizontal gene transfer from animal pathogens. Studies on the molecular mechanism of resistance gene dissemination in different bacterial species of animal origin could provide useful information for preventing or controlling the spread of resistance between animal and human pathogens.

**Electronic supplementary material:**

The online version of this article (10.1186/s13756-018-0415-0) contains supplementary material, which is available to authorized users.

## Background

Florfenicol, which is only used to treat animal infections, is a derivative of chloramphenicol that is active against chloramphenicol-resistant isolates [1]. Resistance to chloramphenicol occurs mainly through the production of inactivating enzymes called chloramphenicol acetyl transferases (CATs) [2] and chloramphenicol exporters, such as CmlA [3]. Over the past decade, most reports have demonstrated that the bacteria causing animal respiratory diseases show high resistance levels to chloramphenicol but are susceptible to florfenicol [4]. However, the resistance levels and number of bacteria that are resistant to florfenicol have increased due to the widespread use of florfenicol in the treatment of animal diseases [5–7]. A study on 1001 bacterial isolates showed that the resistance rates for trimethoprim/sulfamethoxazole and tetracycline were 3.0% and 14.7% in *Actinobacillus pleuropneumoniae* and 6.0% and 81.8% in *S. suis*, respectively, while the resistance rate for florfenicol was < 1% for all strains [8]. Other reports have cited different resistance rates. In Australia, 2.0% and 6.0% of *A. pleuropneumoniae* and *Pasteurella multocida* strains isolated from pig respiratory infections were resistant to florfenicol, respectively [9]. The resistance rate of *E. coli* strains from canine urinary tract infections to florfenicol was higher than that of other pathogens: 31.6% (36/114) [5].

The first florfenicol resistance gene, *pp-flo* (renamed *flo*), was identified on a plasmid in the fish pathogen *Photobacterium damselae* subsp. *piscicida* in 1996 [10]. The *floR* gene is closely related (97% identity) to the *flo* gene [11], and their proteins share 47% amino acid sequence identity with the CmlA protein. The *floR* gene was first reported in 1999 on the chromosome of the worldwide epidemic strain *Salmonella enterica* serovar Typhimurium DT104 [11]. The primary source of human DT104 infections was thought to be animal populations, with both direct contact and foodborne modes of transmission [12]. The IncC plasmid R55, which was initially described to be capable of conferring non-enzymatic chloramphenicol resistance in the 1970s, was then identified in *Klebsiella pneumoniae* [13]. Currently, nine florfenicol resistance genes [*floR*, *floRv*, *floSt*, *fexA*, *fexB*, *pexA*, *cfr, optrA* and *estDL136*] have been identified. With the exception of *cfr* and *estDL136*, which encode a 23S rRNA methyltransferase and a hydrolase, respectively, all of the genes encode exporters [14–18]. The *floR* gene and its analogs have mainly been identified in gram-negative bacteria, whereas the other resistance genes have mainly been detected in gram-positive bacteria [15–17].

Similar to other resistance genes, *floR* has been identified on both chromosomes and plasmids and has often been associated with mobile genetic elements and genomic islands [19, 20]. Mobile genetic elements enable translocation of the *floR* gene between DNA molecules, such as chromosomes and plasmids. A plasmid carrying the *floR* gene can spread among bacteria of the same and different species or genera via conjugation or transformation, thereby disseminating resistance [21]. Bacteria generally obtain multiple resistance genes through the horizontal transfer of plasmids carrying resistance genes [22].

*K. pneumoniae*, which is a member of the *Enterobacteriaceae*, is an opportunistic pathogen for both animals and humans. This bacterium is pervasive in the natural environment and benignly colonizes the gastrointestinal tracts of healthy humans and animals. However, the bacterium is also capable of causing a wide range of diseases in humans and different animal species [23]. *K. pneumoniae* strains are a common cause of health-care associated infections including pneumonia, urinary tract infections (UTIs), and bloodstream infections for critically ill and immunocompromised patients. These strains also infect healthy people in community settings, causing severe infections including pyogenic liver abscess, endophthalmitis, and meningitis [24]. For example, in animals, *K. pneumoniae* strains are well documented to cause mastitis and wounds in cattle [25]; endometritis, cystitis, and liver abscess in horses; tracheitis and wounds in birds; cystitis, phlebitis and otitis externa in dogs; and cystitis in cats [26]. *K. pneumoniae* has also been associated with classical foodborne disease outbreaks [19]. Notably, the prevalence of antibiotic resistance is increasing among *Enterobacteriaceae*, including *K. pneumoniae* [23, 27]. In this study, we used multiple genetic approaches to investigate the *floR* gene in *K. pneumoniae* isolates of human origin and to further demonstrate the potential transmission of this resistance determinant between animal and human pathogens.

## Methods

### Bacterial strains

The 328 non-duplicate *K. pneumoniae* strains used in this work were isolated from patient samples at the First Affiliated Hospital of Wenzhou Medical University in Wenzhou, China, from 2010 to 2014. This sample set included all *K. pneumoniae* strains collected during this time frame. Among these isolates, 55 were isolated in 2010, 109 in 2011, 55 in 2013 and 109 in 2014. The strains were identified using the Vitek-60 microorganism auto-analysis system (BioMerieux Corporate, Craponne, France).

### PCR amplification of the *floR* gene

Total genomic DNA was extracted from the 328 isolates using AxyPrep Bacterial Genomic DNA Miniprep kits (Axygen Scientific, Union City, CA, USA). Template DNA was screened for the *floR* gene using a PCR method. According to the conserved *floR* gene-related regions of the *K. pneumoniae* genome obtained from a pool of strains mainly from this work [28], *floR* gene screening primers were designed and named P_SCR-F_ and P_SCR-R-A/G_, which correspond to the cm101 and cm115 primer sequences, respectively [29]. The sequence of the forward primer P_SCR-F_ was 5’-TTTGGTCCGCTCTCAGAC-3′. Two variants of the reverse primer were used due to a single nucleotide polymorphism (A/G) identified in the region where the primer was designed: 5’-CGAGAAGAAG**A**CGAAGAAG-3′ (P_SCR-R-A_) and 5’-CGAGAAGAAG**G**CGAAGAAG-3′ (P_SCR-R-G_). These primers yield a product 496 bp in length. PCR amplification was carried out under the following conditions: an initial denaturation of 5 min at 95 °C; 35 cycles of denaturation (94 °C for 45 s), annealing (58 °C for 45 s), and extension (72 °C for 90 s); and a final extension step at 72 °C for 10 min [29]. The PCR products were purified using a MinElute PCR Purification kit (QIAGEN China, Shanghai, China) and sequenced by Sanger sequencing (in this work, all the PCR products and cloned fragments were sequenced by Sanger sequencing). The nucleotide sequences were analyzed and compared using the BLAST program (http://www.ncbi.nlm.nih.gov/BLAST).

### Antimicrobial susceptibility testing

Antimicrobial susceptibility testing performed via the agar dilution method in accordance with the guidelines of the Clinical and Laboratory Standards Institute (CLSI document M100-S27, 2017) was used to determine the minimum inhibitory concentrations (MICs) [30]. The resistance threshold values (32 μg/mL) for both chloramphenicol and florfenicol were chosen according to the guidelines of CLSI document M100-S27 (2017) [30] and a publication for *E. coli* [31], respectively. *E. coli* ATCC 25922 was used as a quality control strain.

### Pulsed-field gel electrophoresis (PFGE)

To assess the epidemiology of clinical isolates with *floR* genes, genomic DNA from *K. pneumoniae* isolates harboring *floR* genes was prepared for PFGE typing and digested with 40 U of *Xba* I (Takara, Dalian, China). The protocol and the *Xba* I restriction patterns of genomic DNA from the isolates were analyzed and interpreted according to initial criteria [32]. The Bio-Rad Quantity One program was used to analyze the PFGE results, and a minimum spanning tree was constructed using a categorical coefficient with unweighted pair group method with arithmetic mean (UPGMA) clustering [33].

### Plasmid DNA extraction and sequencing

For plasmid (pKP18–125) sequencing, the transconjugant KP18/EC600 was incubated overnight in 5 mL of Luria-Bertani broth at 37 °C for approximately 16 h to an optimum optical density (OD_600_) of 1.5 ± 0.2. The plasmid was then extracted using the alkaline lysis method as described previously [34]. Plasmid DNA was sequenced via Illumina HiSeq-2000 and Pacific Bioscience sequencing methods at the Beijing Genomics Institute (Beijing, China). Reads derived from the HiSeq-2000 sequencing were initially assembled de novo using SOAPdenovo software to obtain contigs of the plasmid. Pacific Bioscience sequencing reads of approximately 10–20 kb in length were mapped onto the primary assembly to scaffold the contigs. The gaps were filled either by remapping the short reads from HiSeq-2000 sequencing or by PCR product sequencing of the gaps. Glimmer software was used to predict protein-coding genes with potential open reading frames (ORF) > 150 bp [35]. Gview was used to construct basic plasmid features [36]. BLASTX was used to annotate the predicted protein-coding genes against the non-redundant protein database using an e-value threshold of 1e-5.

### Collection and processing of *floR* gene-related sequences

In addition to the pKP18–125 sequence, other sequences containing the *floR* gene were obtained from the NCBI nucleotide database using *floR*, *pp-flo*, *flo*, *cmlA-like, floRv* and *floSt* as key terms. The resulting sequences were filtered, and only sequences containing a complete *floR* gene more than 9 kb in length (with approximately 4 kb both upstream and downstream of the *floR* gene) were retained. Multiple sequence alignments were performed using mafft with the 9-kb *floR* gene-related fragment of pKP18–125 (KY082186) as a reference [32], and the sequences were clustered with an identity of 80%. The sequence with greatest similarity to the other sequences in each cluster was chosen as a candidate for orthologous analysis. Orthologous groups of genes from the candidate sequences were identified using BLASTP and InParanoid [37]. The sequence retrieval, statistical analyses and other bioinformatics tools used in this study were accomplished using Python and Biopython scripts [38].

### Cloning experiments

To identify and clone *floR* genes, we PCR amplified a fragment including the *floR* ORF sequence and its upstream 354-bp potential promotor region from strains positive for the *floR* gene. A set of PCR primers (P_ORF-F_ and P_ORF-R_) was designed using the *K. pneumoniae* plasmid pR55 sequence (JQ010984.1) as a reference. The primer sequences of P_ORF-F_ and P_ORF-R_ were 5’-GTCGAGAAATCCCATGAGTTCA-3′ and 5’-CAGACAGGATACCGACATTCAC-3′, respectively. The PCR products were eluted from agarose gels and ligated into the pMD18 vector (TaKaRa, Dalian, China). Each recombinant plasmid (pMD18-*floR*) was transformed into *E. coli* JM109 using the calcium chloride method, after which the bacterial colonies were grown on Luria-Bertani agar plates supplemented with ampicillin (100 μg/mL). The recombinant plasmids were isolated and digested with restriction enzymes to confirm insertion of a *floR* fragment of approximately 1600 bp in length. Each cloned *floR* fragment was analyzed by Sanger sequencing from a purified transformant and was further compared to the reference *floR* gene (JQ010984.1) using the BLASTN program.

### Conjugation experiments

To examine the conjugation potential of resistance gene-harboring pKP18–125, we used rifampin-resistant EC600 as a recipient strain in a biparental mating, which was performed overnight at 37 °C on sterile nitrocellulose filters as previously described [39]. The transconjugants were selected on Mueller-Hinton agar plates containing 1200 μg/mL of rifampin and 16 μg/mL of florfenicol [40]. Plasmid DNA was extracted from transconjugants, and the presence of the *floR* gene was verified by PCR and PCR product sequencing. The plasmid (pKP18–125) of one transconjugant (KP18/EC600) was sequenced in full to verify that the *floR* gene was encoded on this transferable plasmid.

## Results

### *floR* gene detection and sequencing

Approximately 7.01% (23/328) of the isolates were positive for *floR* (Table 1). Of the 23 *floR*-positive strains, 4, 8, 4 and 7 strains were isolated in 2010, 2011, 2013 and 2014, respectively. The positive rates were similar among the strains collected from different years (7.27% [4/55] in 2010, 7.34% [8/109] in 2011, 7.27% [4/55] in 2013 and 6.42% [7/109] in 2014). Twenty-two fragments containing the *floR* ORF and their upstream potential promotor regions were successfully cloned; all the cloned ORF sequences shared approximately 99% nucleotide sequence identity. No amino acid variants were identified in the cloned ORFs.

**Table 1:** Strains and plasmids used in this study

Strain or plasmid	Relevant characteristic(s)^a^	Reference or source
Strain		
KP1 - KP23	23 strains carrying the *floR* gene from 328 clinically isolated *K. pneumoniae* samples	this study
JM109	*Escherichia coli* JM109 was used as a host for the PCR product cloning	
EC600	*Escherichia coli* C600 *was* used as a host in conjugation experiments; Rf^**r**^	
ATCC25922	*Escherichia coli* ATCC25922 is an FDA clinical isolate	
*E. coli* carrying plasmid		
pMD18-*floR*s/JM109	JM109 carrying the pMD18 vector encoding *floR* gene regions from 22 *floR* gene-positive strains	This study
pKP5/EC600	The transconjugant with KP5 plasmid transferred into EC600	This study
pKP6/EC600	The transconjugant with KP6 plasmid transferred into EC600	This study
pKP18/EC600	The transconjugant with KP18 plasmid transferred into EC600	This study
Plasmid		
pKP18–125	KP18 plasmid transferred into EC600 by conjugation and sequenced	This study
pMD18	Cloning vector for the PCR products of *floR* genes; Ap^**r**^	TaKaRa

^a^*Rf* rifampin, *Ap* ampicillin

### Florfenicol and chloramphenicol MICs of the strains

The MICs of florfenicol and chloramphenicol were determined for the 328 clinical isolates, 3 transconjugants and transformants with cloned *floR* genes. The MIC results showed that 57 of the 328 clinical isolates (17.38%) were resistant to both florfenicol and chloramphenicol, whereas 67 (20.42%) and 113 (34.45%) of the strains were resistant to florfenicol and chloramphenicol, respectively. A total of 7.62% (25/328) and 11.59% (38/328) of the strains were resistant to florfenicol and chloramphenicol, respectively, with MIC values ≥512 μg/mL, and 64.93% (213/328) of the strains were susceptible, with MIC values < 32 μg/mL for both antibacterial agents.

The strains positive for the *floR* gene had much higher MIC values for both florfenicol and chloramphenicol than the *floR*-negative strains. Of the 23 *floR-*positive strains, 95.65% (22/23) showed high MIC values to florfenicol (≥512 μg/mL) (Table 2). Among the 305 strains negative for the *floR* gene, only 14.43% of the strains (44/305) showed resistance to florfenicol, and only 1.64% (5/305) of the strains had MIC values ≥512 μg/mL. The MIC values between the transformants with cloned *floR* genes and the clinical isolates were similar (Table 2).

**Table 2:** MIC values for the *floR-*positive *K. pneumoniae* strains, transformants expressing cloned *floR* genes and transconjugants (μg/mL)

	Florfenicol			Chloramphenicol		
Name	Clinical isolate	Transformant	Transconjugant	Clinical isolate	Transformant	Transconjugant
KP5	1024	512	512	256	128	512
KP6	1024	256	512	512	256	512
KP18	> 1024	512	512	512	128	256
KP23	64			256		
KP4, 14, 19, 22	512			256		
KP21	512			128		
KP3, 8, 15	1024			1024		
KP10, 12, 13	1024			256		
KP11, 20	1024			128		
KP2, 7, 9, 16, 17	> 1024			> 1024		
KP1	> 1024			512		
ATCC25922	4					
JM109	4					
EC600	4					

### A transferable plasmid carrying the *floR* gene

The results of the conjugation experiments for the 23 *floR*-positive clinical isolates showed that plasmids in three donor strains (KP5, KP6 and KP18) were successfully transferred to the recipient. PFGE analysis showed that the wild-type donors KP5, KP6 and KP18 harbored 2, 4 and 2 plasmids, respectively. However, each of the transconjugants (KP5/EC600, KP6/EC600 and KP18/EC600) only harbored the largest plasmid of the donor cells. The results of PCR product sequencing confirmed that *floR* genes were located on the transferred plasmids. The MIC results showed that the florfenicol and chloramphenicol resistance levels of the transconjugants were similar to those of the donor strains (Table 2).

### Clonal relatedness of the *floR*-positive *K. pneumoniae* strains identified by PFGE

**Fig. 1:**
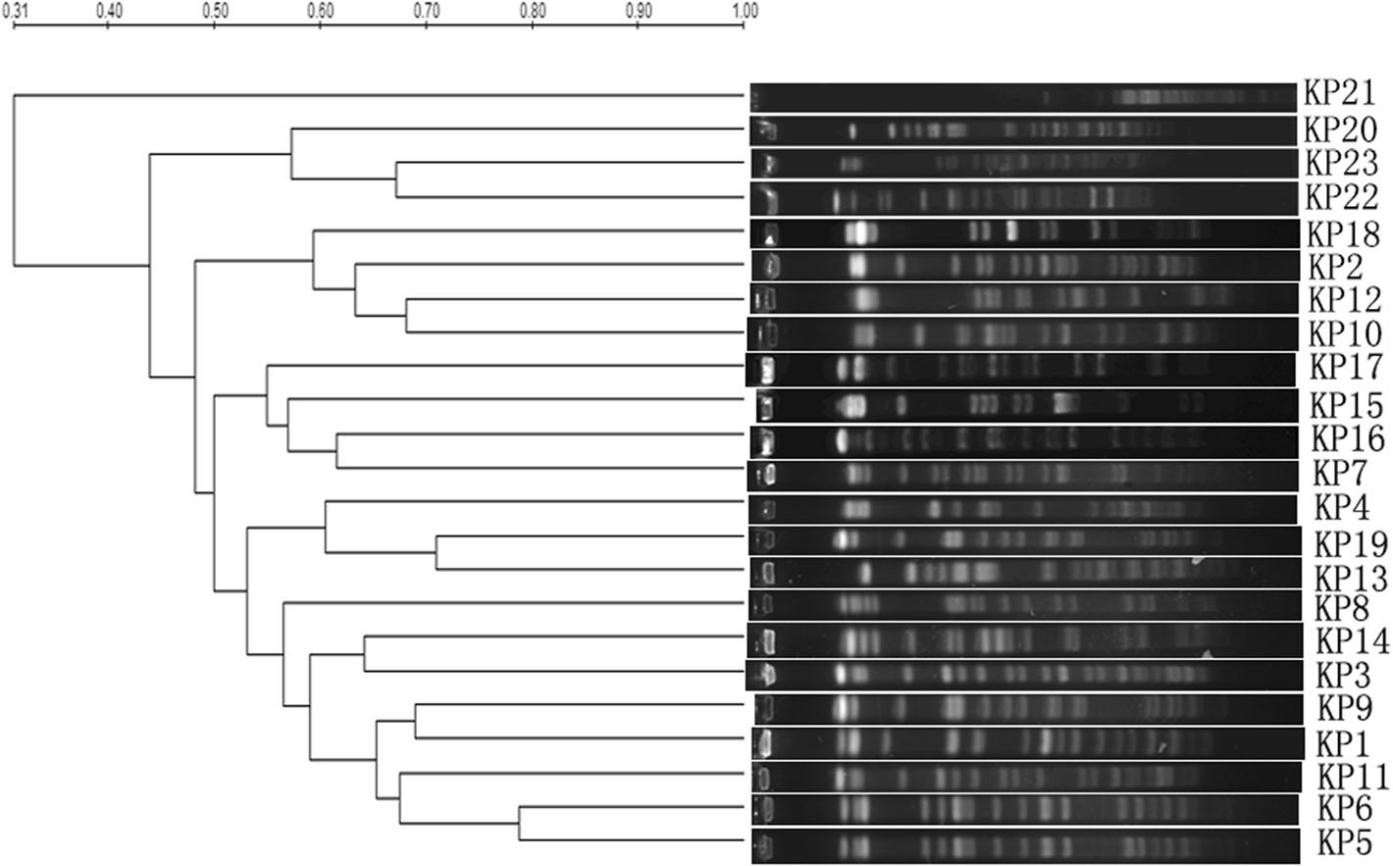
Pulsed-field gel electrophoresis of *Xba*I-digested genomic DNA from the 22 *floR*-positive *K. pneumoniae* strains. Only one cluster, composed of KP5 and KP6, was determined to be clonally related

PFGE patterns with ≥80% identity were interpreted as closely or possibly related to the outbreak isolates. Of the 23 strains detected, 22 had good fingerprints; one strain (KP21) without clear bands could not be compared. Only two strains (KP5 and KP6) showed similar fingerprint patterns, whereas the remaining 20 strains had different genotypes (Fig. 1). KP5 was isolated from a sputum sample of a male patient in the Department of Neurosurgery in March 2014, while KP6 came from a sputum sample of a female patient in the intensive care unit (ICU) in the Department of Brain Surgery in April 2014.

### Structure and comparative genomics analysis of *floR* gene-related regions

**Fig. 2:**
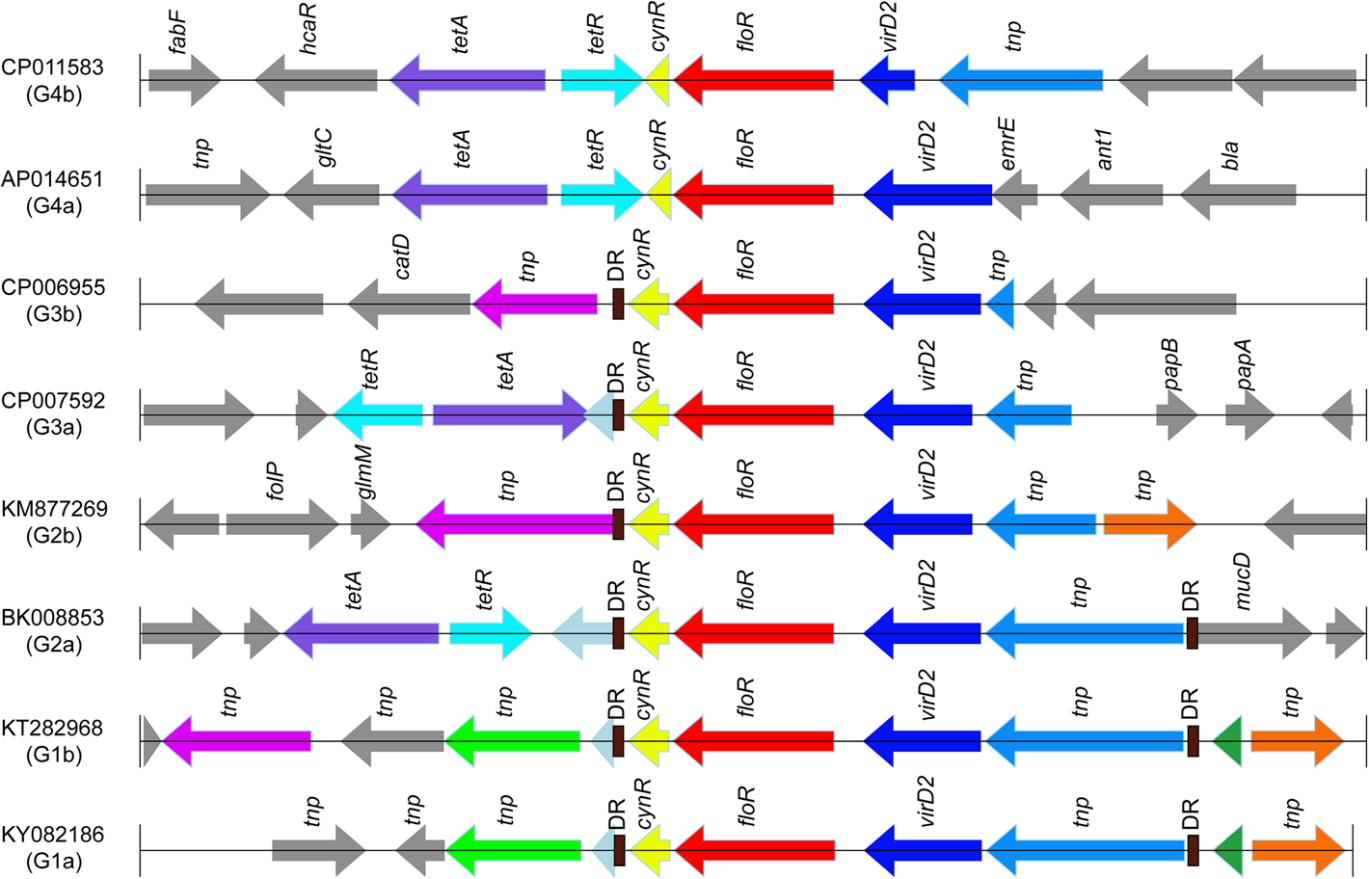
Structure of the *floR* gene-related regions. Eight representative sequences from the four groups (one from each subgroup) are presented with their accession numbers. The arrows represent sequence units, and the same units are shown in the same color. The names of the sequence units are indicated over the arrows, with the sequence units of unknown function left blank

pKP18–125 was 125,329 bp in length. Annotation determined that the plasmid carried one replicon belonging to incompatibility group FII (IncFII) and harbored 164 coding sequences (CDs). The plasmid genome can be divided into 4 regions according to the functions of the ORFs as follows: the variable region, the conjugation region, the transfer leading region and the replication region. The variable region is approximately 40 kb in length and encodes 42 ORFs, including approximately 20 genes related to drug resistance, 13 recombination-related genes or structures (i.e., integrase and transposase genes and insertion sequences [ISs]) and 9 genes of unknown function. According to the structure of the mobile genetic elements, this region could be roughly divided into six units, including one class 1 integron and five transposons. The *floR* gene was located in a transposon-like fragment approximately 9 kb in length (accession number: KY082186) consisting of a conserved gene cluster of *lysR*-*floR*-*virD2*, 5 *tnp* units and two direct repeats (DRs). In this work, we mainly analyzed the structural characteristics of the 9-kb *floR* gene-related transposon-like fragment (Fig. 2).

Overall, a total of 105 DNA sequences of approximately 9 kb in length with the *floR* gene in their center were retrieved from all *floR* gene-containing sequences in the NCBI nucleotide database. Of these sequences, 45 were from complete or partial bacterial chromosomes, and 60 were from plasmid sequences. Through a multiple sequence alignment, 27 clusters with identities greater than 80% were obtained. According to the similarity of the core sequences adjacent to the *floR* gene, the sequences of these 27 clusters were orthologously analyzed and further clustered into 4 groups (G1- G4), with each group containing 2 subgroups (e.g., G1a and G1b). Group 1 consisted of only 2 sequences (KY082186 and KT282968) that shared approximately 7 kb in common (Fig. 1 and Table S1).

Eight representative sequences from the eight subgroups (one from each subgroup) are illustrated in Fig. 2 with their accession numbers. Sequences similar to the representative sequences are shown in Additional file 1: Table S1. The results of this orthologous analysis revealed that an approximately 2-kb sequence encoding *lysR*-*floR*-*virD2* was conserved and present in the majority of the sequences (79.0%, 83/105). Many of these sequences also shared the same upstream DR and complete or truncated downstream *tnp* unit (Fig. 1). The 9-kb *floR* gene-related transposon-like fragment of pKP18–125 in this study showed highest similarity to a 7-kb fragment from the plasmid pEC012 (KT282968). Interestingly, although pKP18–125 was isolated from a *K. pneumoniae* strain from a patient in South China, pEC012 was found in an *E. coli* isolate from a chicken in North China [41].

## Discussion

In this study, we found that among all the clinical *K. pneumoniae* isolates detected, 20.42% (67/328) were resistant to florfenicol, of which 7.01% (23/328) carried the *floR* gene, but 13.41% (44/328) were free of the *floR* gene. A similar report demonstrated a *floR* gene positivity rate of only 21.8% (26/119) among 119 florfenicol-resistant gram-negative bacilli from several freshwater Chilean salmon farms [42]. Our MIC results for the 328 strains demonstrated that the *floR* gene played a key role in the resistance of these bacteria to florfenicol. The *floR*-positive strains had a much higher resistance rate (23/23, 100%) and much higher MIC values for florfenicol (22/23, 95.65% with MIC values ≥512 μg/mL) than the *floR*-negative strains, which had a resistance rate of 14.43% (44/305) with only 1.64% (5/305) of the strains having MIC values ≥512 μg/mL. At present, of the nine florfenicol resistance genes*,* the *floR* gene is the only known florfenicol resistance gene that has been identified in *K. pneumoniae* strains of either human or animal origin [43]. Five genes (*fexA*, *fexB*, *pexA*, *optrA* and *cfr*) were mainly identified in gram-positive bacteria [15–17]. The *cfr* gene has also been occasionally identified in *E. coli* or *Proteus vulgaris* [44, 45] and *fexA* and *pexA* were once identified in *E. coli* [44]. The other three genes have only been identified in certain gram-negative bacteria (*floRv* in *Stenotrophomonas maltophilia* [46], *floSt* in *Salmonella* [47] and *estDL136* in *E.coli* [44]). We hypothesize that other mechanisms, such as exporters and enzymes, in addition to the known florfenicol resistance genes, may also be responsible for florfenicol resistance in gram-negative bacteria including *K. pneumoniae*.

The *floR* genes were located on both chromosomes and plasmids amidst various mobile genetic elements, indicating that horizontal transfer of the *floR* gene occurred among bacteria of different species. The *floR* gene was identified first on the chromosome of *S. typhimurium* DT104 (*Salmonella typhimurium* DT104) and then on a plasmid of *E. coli* isolate BN10660 [48] and was also identified on the IncC plasmid R55 harbored by *K. pneumoniae* [13] and on other sources [17, 43]. In *S. typhimurium* DT104, the *floR* gene was included in a 12.5-kb region with multiple resistance genes. The *tetR* and *tetA* tetracycline resistance genes were located downstream of the *floR* gene and were flanked by two integrons. One integron contained an *aadA2* gene and an incomplete *sulI* resistance gene, and the other harbored a β-lactamase gene and a complete *sulI* gene [49]. In pKP18–125, the downstream region was a class 1 integron that contained 5 resistance genes (*acc(6′)*, *arr2*, etc.) and was different from the 12.5-kb region of the *S. typhimurium* DT104 chromosome. Interestingly, the sequence most similar to the *floR*-containing fragment on pKP18–125 from a clinical *Klebsiella pneumoniae* isolate was located on pEC012 (KT282968), a plasmid from an *E. coli* strain isolated from a chicken [50]. This finding suggests that horizontal transfer of the *floR*-containing fragment occurred between bacteria of animal and human origins.

Our PFGE analysis revealed that two *floR*-positive strains (KP5 and KP6) had similar PFGE profiles. They were isolated from the same sample type (sputum) but were found in different hospitalized patients during different time periods. Some *K. pneumoniae* strains carrying resistance genes were previously reported to have caused outbreaks in European countries, indicating the potential risk of the spread of resistance genes through bacterial outbreaks, especially those caused by bacteria with resistance plasmids [51]. Although the relationship between the two strains carrying *floR* is still in question, effort should be made to avoid any pathogen outbreaks in hospital environments.

## Conclusions

Our study demonstrated that 20.42% (67/328) of the clinical *K. pneumoniae* isolates were resistant to florfenicol, but only 7.01% (23/328) carried the *floR* gene. The *floR* gene was related to a transposon-like sequence and located on a conjugative plasmid. The most similar sequence to the *floR*-containing fragment on pKP18–125 was that a fragment on pEC012 in an *E. coli* strain isolated from a chicken. This finding indicates that resistance genes in animal pathogens might be disseminated to human pathogens. The dissemination of resistance genes from animals to humans reflects the risk to human health of antimicrobial agent use in animals. In addition, these results highlight the critical need to consistently implement effective strategies to prevent transmission and infection. Combating antibiotic-resistant bacteria supports patient care, agriculture, economic growth and national security.

## Additional file

Additional file 1: Table S1 Grouping of 105 *floR* gene containing sequences and their origins. (PDF 67 kb)

## Abbreviations

Ap: Ampicillin
BLAST: Basic local alignment search tool
ICU: Intensive care unit
MICs: Minimum inhibitory concentrations
PFGE: Pulsed-field gel electrophoresis
Rf: Rifampin
