# Supplementary material for: Spread of the florfenicol resistance floR gene among clinical Klebsiella pneumoniae isolates in China
Source: Antimicrob Resist Infect Control. 2018 Nov 1;7:127. doi: 10.1186/s13756-018-0415-0 (PMC6211440; doi:10.1186/s13756-018-0415-0)
Supplement: Supplementary file 1 — Table S1 Grouping of 105 floR gene containing sequences and their origins. (PDF 67 kb) [file 13756_2018_415_MOESM1_ESM.pdf]

Supplementary table 1 Grouping of 105 *floR* gene containing sequences and their origins

Accession number	Group	Cluster	Bacteria	Location
KY082186	G1a	1	*Klebsiella pneumoniae*	plasmid
KT282968.1	G1b	2	*Escherichia coli*	plasmid
KT151662.1	G2a	4	*Vibrio cholerae*	chromosome
KJ817376.1	G2a	4	*Vibrio cholerae*	chromosome
KT151654.1	G2a	4	*Vibrio cholerae*	chromosome
KT151656.1	G2a	4	*Vibrio cholerae*	chromosome
KT151657.1	G2a	4	*Vibrio cholerae*	chromosome
KT151658.1	G2a	4	*Vibrio cholerae*	chromosome
KT151659.1	G2a	4	*Vibrio cholerae*	chromosome
KT151661.1	G2a	4	*Vibrio cholerae*	chromosome
KT151655.1	G2a	4	*Vibrio cholerae*	chromosome
KR091911.1	G2a	7	*Salmonella enterica*	plasmid
CP007137.1	G2a	7	*Escherichia coli*	plasmid
CP006029.1	G2a	7	*Escherichia coli*	plasmid
CP009413.2	G2a	7	*Salmonella enterica*	plasmid
JN983043.1	G2a	7	*Salmonella enterica*	plasmid
CP009567.1	G2a	7	*Salmonella enterica*	plasmid
CP009570.1	G2a	7	*Salmonella enterica*	plasmid
AB571791.1	G2a	7	*Salmonella enterica*	chromosome
AP014565.1	G2a	7	*Salmonella enterica*	chromosome
CP012682.1	G2a	7	*Salmonella enterica*	plasmid
CP006641.1	G2a	8	*Escherichia coli*	plasmid
KF787110.1	G2a	8	*Escherichia coli*	plasmid
CP009042.1	G2a	10	*Vibrio cholerae*	chromosome
KJ909292.1	G2a	10	*Aeromonas salmonicida*	plasmid
GQ463139.1	G2a	10	*Providencia alcalifaciens*	chromosome
GQ463140.1	G2a	10	*Vibrio cholerae*	chromosome
AB114188.1	G2a	10	*Vibrio cholerae*	chromosome
GQ463141.1	G2a	10	*Vibrio cholerae*	chromosome
GQ463142.1	G2a	10	*Vibrio cholerae*	chromosome
AB535680.1	G2a	10	*Vibrio cholerae*	chromosome
CP003069.1	G2a	10	*Vibrio cholerae*	chromosome
CP007634.1	G2a	10	*Vibrio cholerae*	chromosome
AY034138.1	G2a	10	*Vibrio cholerae*	chromosome
JN648379.1	G2a	10	*Vibrio cholerae*	chromosome
KC886257.1	G2a	10	*Vibrio cholerae*	chromosome
AY055428.1	G2a	10	*Vibrio cholerae*	chromosome
KC886258.1	G2a	10	*Vibrio cholerae*	chromosome
CP009414.2	G2a	11	*Salmonella enterica*	plasmid
KJ909290.1	G2a	11	*Aeromonas salmonicida*	plasmid
KC853434.1	G2a	11	*Escherichia coli*	plasmid
HQ023863.1	G2a	11	*Escherichia coli*	plasmid

FJ621588.1	G2a	11	*Escherichia coli*	plasmid
FJ621586.1	G2a	11	*Escherichia coli*	plasmid
HQ023861.1	G2a	11	*Escherichia coli*	plasmid
KP056256.1	G2a	11	*Escherichia coli*	plasmid
HQ023862.1	G2a	11	*Escherichia coli*	plasmid
JX442976.1	G2a	11	*Klebsiella pneumoniae*	plasmid
JN687470.1	G2a	11	*Providencia stuartii*	plasmid
FJ621587.1	G2a	11	*Salmonella enterica*	plasmid
CP009410.2	G2a	11	*Salmonella enterica*	plasmid
CP009411.2	G2a	11	*Salmonella enterica*	plasmid
CP009412.2	G2a	11	*Salmonella enterica*	plasmid
JF267651.1	G2a	11	*Salmonella enterica*	plasmid
JN983045.1	G2a	11	*Salmonella enterica*	plasmid
JN983048.1	G2a	11	*Salmonella enterica*	plasmid
CP009409.2	G2a	11	*Salmonella enterica*	plasmid
CP009563.1	G2a	11	*Salmonella enterica*	plasmid
CP009564.1	G2a	11	*Salmonella enterica*	plasmid
CP009560.1	G2a	11	*Salmonella enterica*	plasmid
CP009562.1	G2a	11	*Salmonella enterica*	plasmid
CP000604.1	G2a	11	*Salmonella enterica*	plasmid
AB591424.1	G2a	11	*Salmonella enterica*	plasmid
CP011429.1	G2a	11	*Salmonella enterica*	plasmid
CP007636.1	G2a	11	*Vibrio cholerae*	plasmid
BK008853.1	G2a	16	*TPA_inf: Aeromonas*	plasmid
EF495198.1	G2a	16	*Aeromonas bestiarum*	plasmid
LN735558.1	G2b	3	*Escherichia coli*	plasmid
LN735561.1	G2b	3	*Escherichia coli*	plasmid
LN735559.1	G2b	3	*Escherichia coli*	plasmid
KM877269.1	G2b	6	*Salmonella enterica*	plasmid
FJ012880.1	G2b	6	*Uncultured bacterium*	plasmid
NG_035718.1	G2b	19	*Escherichia coli*	plasmid
AB571865.1	G2b	22	*Photobacterium damselae*	plasmid
LC055503.1	G3a	9	*Klebsiella pneumoniae*	plasmid
KF250428.1	G3a	9	*Klebsiella pneumoniae*	plasmid
CP007592.1	G3a	12	*Escherichia coli*	chromosome
CP006955.1	G3a	17	*Bibersteinia trehalosi*	plasmid
CP003022.1	G3a	21	*Pasteurella multocida*	chromosome
CP003745.1	G3b	18	*Bibersteinia trehalosi*	plasmid
CP006956.1	G3b	20	*Bibersteinia trehalosi*	chromosome
JQ010984.1	G3b	23	*Klebsiella pneumoniae*	plasmid
KP276584.1	G3b	23	*Escherichia coli*	plasmid
CP011374.1	G4a	15	*Moraxella bovoculi*	chromosome
CP006657.1	G4a	25	*Klebsiella pneumoniae*	plasmid
CP003225.1	G4a	25	*Klebsiella pneumoniae*	plasmid

AP014651.1	G4a	25	*Pseudomonas aeruginosa*	chromosome
CT025832.1	G4a	27	*Acinetobacter baumannii*	chromosome
KM649682.1	G4b	5	*Stenotrophomonas maltophilia*	chromosome
CP011649.1	G4b	13	*Enterobacter cloacae*	plasmid
CP011580.1	G4b	13	*Enterobacter cloacae*	plasmid
CP011583.1	G4b	14	*Enterobacter cloacae*	plasmid
CP011571.1	G4b	14	*Enterobacter cloacae*	plasmid
KM234279.1	G4b	24	*Salmonella enterica*	chromosome
KJ186152.1	G4b	24	*Proteus mirabilis*	chromosome
KJ186153.1	G4b	24	*Proteus mirabilis*	chromosome
JX121641.1	G4b	24	*Proteus mirabilis*	chromosome
JX121639.1	G4b	24	*Proteus mirabilis*	chromosome
GQ388247.1	G4b	24	*Pseudomonas aeruginosa*	chromosome
KJ463833.1	G4b	24	*Pseudomonas aeruginosa*	chromosome
AY963803.6	G4b	24	*Salmonella enterica*	chromosome
LN829403.1	G4b	24	*Salmonella enterica*	chromosome
AF261825.2	G4b	24	*Salmonella enterica*	chromosome
CP007581.1	G4b	24	*Salmonella enterica*	chromosome
CU459141.1	G4b	26	*Acinetobacter baumannii*	chromosome
